# Population structure and genetic diversity of non-native aoudad populations

**DOI:** 10.1038/s41598-021-91678-2

**Published:** 2021-06-10

**Authors:** Sunčica Stipoljev, Toni Safner, Pavao Gančević, Ana Galov, Tina Stuhne, Ida Svetličić, Stefano Grignolio, Jorge Cassinello, Nikica Šprem

**Affiliations:** 1 Department of Fisheries, Apiculture, Wildlife Management and Special Zoology, Faculty of Agriculture, University of Zagreb, 10000 Zagreb, Croatia; 2 Department of Plant Breeding, Genetics and Biometrics, Faculty of Agriculture, University of Zagreb, 10000 Zagreb, Croatia; 3 Centre of Excellence for Biodiversity and Molecular Plant Breeding (CroP-BioDiv), 10000 Zagreb, Croatia; 4 Department of Biology, Faculty of Science, University of Zagreb, 10000 Zagreb, Croatia; 5 Department of Veterinary Medicine, University of Sassari, 07100 Sassari, Italy; 6 Estación Experimental de Zonas Áridas (EEZA-CSIC), Carretera de Sacramento s/n, La Cañada de San Urbano, 04120 Almería, Spain

**Keywords:** Population genetics, Biodiversity, Invasive species

## Abstract

The aoudad (*Ammotragus lervia* Pallas 1777) is an ungulate species, native to the mountain ranges of North Africa. In the second half of the twentieth century, it was successfully introduced in some European countries, mainly for hunting purposes, i.e. in Croatia, the Czech Republic, Italy, and Spain. We used neutral genetic markers, the mitochondrial DNA control region sequence and microsatellite loci, to characterize and compare genetic diversity and spatial pattern of genetic structure on different timeframes among all European aoudad populations. Four distinct control region haplotypes found in European aoudad populations indicate that the aoudad has been introduced in Europe from multiple genetic sources, with the population in the Sierra Espuña as the only population in which more than one haplotype was detected. The number of detected microsatellite alleles within all populations (< 3.61) and mean proportion of shared alleles within all analysed populations (< 0.55) indicates relatively low genetic variability, as expected for new populations funded by a small number of individuals. In STRUCTURE results with K = 2–4, Croatian and Czech populations cluster in the same genetic cluster, indicating joined origin. Among three populations from Spain, Almeria population shows as genetically distinct from others in results, while other Spanish populations diverge at K = 4. Maintenance of genetic diversity should be included in the management of populations to sustain their viability, specially for small Czech population with high proportion of shared alleles (0.85) and Croatian population that had the smallest estimated effective population size (Ne = 5.4).

## Introduction

During the nineteenth and twentieth century, large number of non-native animal species have been widely introduced in Europe. One of the most important drivers for such introductions was hunting, with introductions aiming to create or improve hunting opportunities, especially when native game species had become scarce^1,2^. Newly created populations are often established with relatively few individuals of unknown genetic background and therefore may be particularly susceptible to loss of genetic variation due to inbreeding and genetic drift^3,4^. Within such recently established populations the genetic diversity might decrease, whereas population differentiation might increase over time^5^. Reduced gene flow and the low level of genetic diversity can also enhance the rate of inbreeding, which is considered to reduce a populations’ adaptability to a changing environment. Homozygous recombination of deleterious recessive alleles become more likely, which may lead to inbreeding depression^6^.

Among all introduced groups of mammal species in Europe, ungulates stand out from the others with 73.5%, since this is one of the most important game groups in all European countries^7^. Consequently, both distribution and genetics of ungulate populations across Europe have been profoundly influenced by such introductions^8^. Among ungulates introduced into Europe, the aoudad (*Ammotragus lervia* Pallas 1777) is one of the species that was successfully established in the wild beyond its natural range^8^, with established populations in Croatia, Czech Republic, Italy, and Spain^9^.

The aoudad, or Barbary sheep, is native to the mountain ranges of North Africa. Small groups scattered irregularly on large territories from Mauritania in the west to the Red Sea in the east show a typical pattern of the distribution of the species in the wild^10^. As a result of mostly poaching and habitat loss, population numbers of aoudad in its native range are declining^11,12^, classifying this species as Vulnerable in the IUCN Red List^13^. The aoudad is a generalist herbivore and extremely plastic in its utilization of available food resources^14^. It is a polygynous species with high reproductive rate. Large and strong individuals have high fitness and reproductive success^15^, increasing their potential to colonise different localities whenever conditions are appropriate^11^.

In Croatia, during 2002 a number of aoudads of unknown origin were illegally released in the southern Dinaric region (Mosor Mountain). Current data obtained by GPS tracking of aoudad movements showed that the population is limited to the Mosor Mountain range and it is highly unlikely that it will expand its range beyond that area^16^. The population in the Czech Republic was established following the escape of several individuals from the Plzeň Zoo in 1976, and it consisted of a few dozen animals^17^, while today this population is believed to be extinct (Cupic S., pers. comm.). In Italy, one small population of about 20 individuals is known to be present at the Beigua Regional Park in the province of Savona (Liguria) since 2007^15^. Previously, another population was present in the province of Varese (Lombardy), where a group of six individuals (one male and five females) escaped from a private enclosure in 1993 and established a small breeding population that reached the maximum size of about 20 individuals. This population has been eradicated by the personnel of the Province of Varese accordingly to a plan approved by the Italian National Institute for Environmental Protection and Research (ISPRA) since 2005 (Martinoli A., pers. comm.). In Sierra Espuña, Spain, 36 aoudads, from zoos in Germany and Morocco, were intentionally introduced by the regional administrations in 1970. This founding population reproduced with great success and naturally dispersed very rapidly from their release area to nearby montane enclaves. Its current population, estimated at around 2000 individuals, is still expanding^11,18,19^. In 1972 sixteen animals from Sierra Espuña were released into La Palma island, Canary Islands, and established successfully since then^10,18^.

So far, there is no evidence of the negative impact of the aoudad on host ecosystems in mainland Spain, except for La Palma island, where they have critically affected the survival and diversity of native, endemic flora and caused high levels of soil erosion^11,20^. There is no information on environmental impacts for the other non-native aoudad populations^15^.

When the introduction of species into new areas is human-mediated, the new populations are often founded by only a few individuals that are completely isolated from the source populations. In the case of the European aoudad populations, sudden and substantial reduction in effective population size during first introductions and lack of gene flow into the established populations are assumed to have led to the loss of genetic variation through genetic drift. Severe reductions in genetic diversity may limit viability and adaptive potential of introduced populations under environmental change^5^, as adaptation in such species occurs mainly through selection on pre-existing genetic variation^21^. Since scientific literature about the genetic structure of European aoudad populations is scarce, it is thus important to study genetic diversity and structure of those populations to assess their sustainability. Unfortunately, no comprehensive genetic analyzes have yet been carried out on original aoudad populations from their native range, except for a few studies of specific populations (e.g., Derouiche et al.^22^). In addition, insight into the genetic structure and origin of European aoudad populations might contribute to the ex-situ conservation of the species that is threatened in its native range.

Here we report the first attempt to characterize the genetic diversity and population genetic structure of all non-native European aoudad populations. We used neutral genetic markers, mitochondrial DNA (mtDNA) control region sequence and microsatellite loci, to characterize and compare genetic diversity and spatial pattern of genetic structure on different timeframes among study populations. While variability of maternally inherited and more conserved mtDNA control region reflect maternal lineages present in the founder individuals, microsatellites, due to their codominant nature and high variability can reflect more recent events that shaped current genetic structure^23^. We aimed to understand how current levels of genetic diversity and structuring vary among European aoudad populations that differ with regard to the time and source of introduction.

Based on samples collected from all known locations in Europe, the objectives of this study were to characterize the patterns of neutral genetic structure of recently established non native populations of aoudad and gain insights into the number of maternal lineages of these populations.

## Results

### Mitochondrial sequence analysis

**Figure 1:**
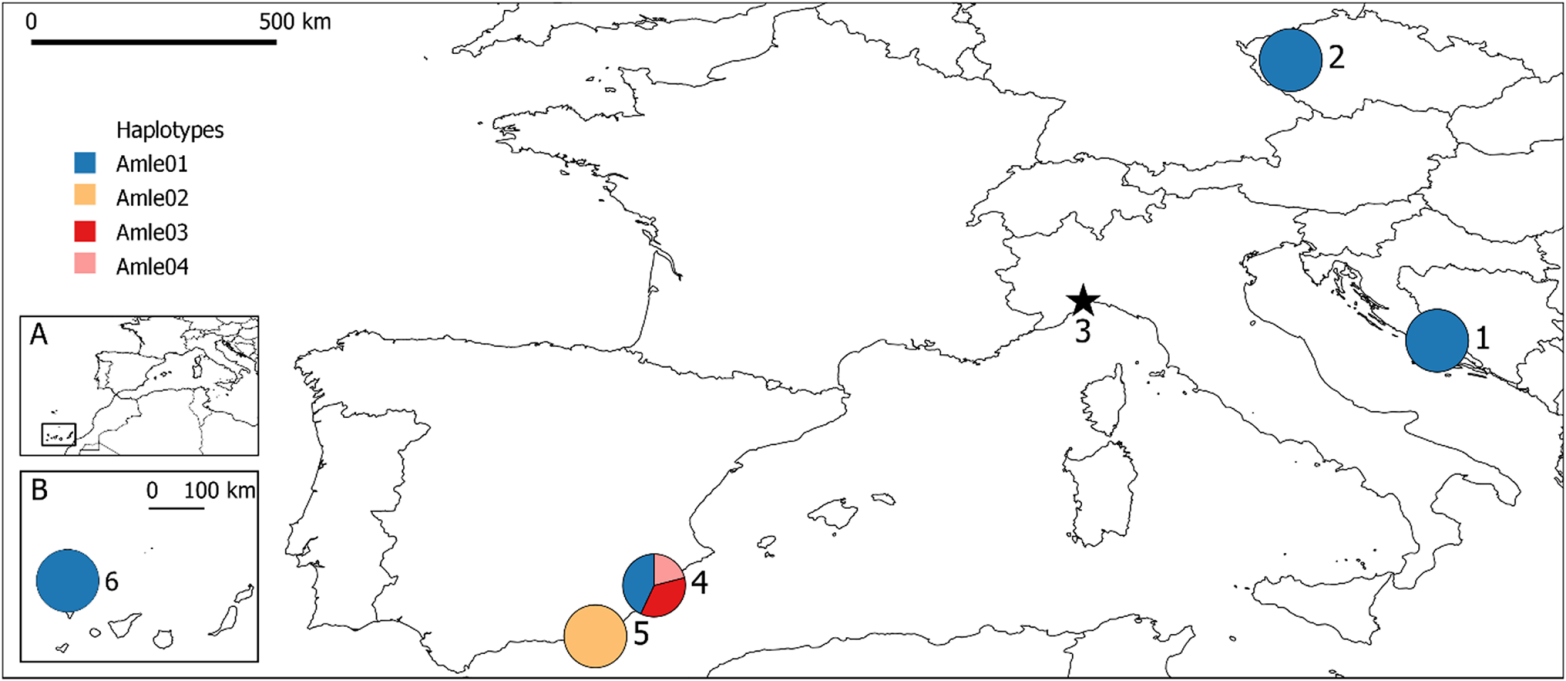
Sampling populations (labelled with a number) and geographic distribution of the four mitochondrial haplotypes identified within study of European aoudad populations. The pie charts indicate the relative frequency of the mtDNA haplotypes in each location: 1—Croatia, Mosor Mountain, 2—Czech, area near city of Plzeň, 3—Italy, Beigua Regional Park (marked with the star, since no haplotypes were revealed due to unsuccessful sequencing), 4—Spain, Sierra Espuña, 5—Spain, Almeria, 6—Spain, La Palma. Inserts show the location of La Palma off the northwestern coast of Africa (**A**), belonging to the archipelago of the Canary Islands (**B**). Map was generated with QGIS 2.18.26 software (QGIS.org, 2021. QGIS Geographic Information System. QGIS Association. http://www.qgis.org).

Sequencing of the mtDNA control region was successfully performed on 69 samples from five different populations (Croatia n = 32, Czech Republic n = 1, Sierra Espuña n = 14, Almeria captivity n = 7, La Palma n = 15). None of the samples collected in Italy were successfully sequenced, due to the low quality of DNA. Low quality of the DNA isolated from faecal samples is known to dramatically reduce the genotyping success of such samples^24^. In the full sample, four mtDNA haplotypes (GenBank accession numbers MW349820–MW349823, Fig. 1) were detected.

**Figure 2:**
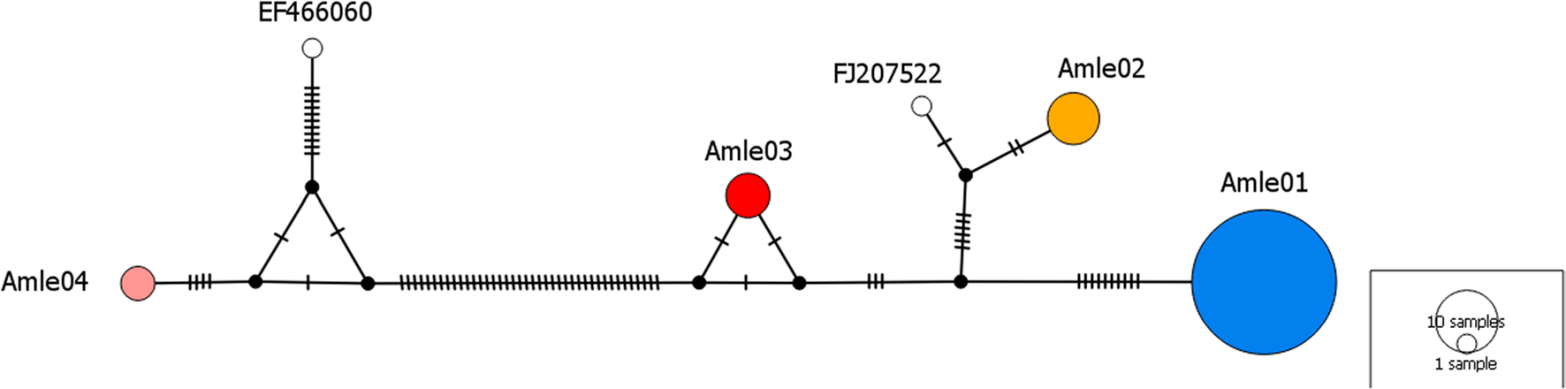
Median‐joining network based on the 606-bp mitochondrial control region haplotypes identified in European aoudad populations. Haplotypes are represented by circles with sizes proportional to the number of individuals. Colors assigned to each haplotype are matching those in Fig. 1, with two haplotypes from GenBank (EF466060 and FJ207522) colored in white. Number of mutations separating nodes is represented by slashes crossed with the network branches.

Haplotype Amle01 was the most frequent (70%) and the only one found in Croatian, Czech and La Palma samples. Samples from Almeria were monomorphic for Amle02 haplotype, therefore haplotype and nucleotide diversity was zero in these populations. Samples from Sierra Espuña comprised of three haplotypes (Amle01, 03 and 04) with haplotype diversity of 0.69 and nucleotide diversity of 0.06. Observed four haplotypes were defined by 59 variable sites of which 11 were parsimony informative. The least number of mutation (12) was present between haplotypes Amle02 and Amle03, while the haplotype Amle04 stood out as remarkably divergent from all others, with the maximum of 55 variable sites separating Amle04 and Amle02 (Fig. 2). Presence of four distinct mtDNA haplotypes in our samples indicates that at least four maternal mtDNA lineages are present in current aoudad populations in Europe.

### Microsatellite genotyping

We succesfully genotyped 85 individuals. Eighty three of them were genotyped at all 15 microsatellite markers, while two samples were genotyped at fourteen and thirteen markers, respectively. In the Croatian population, two multilocus genotypes were shared between four individuals. The unbiased probability of identity, or the probability that two individuals randomly drawn from the population had the same multilocus genotype, was 1.47 × 10^−7^ (Supplementary Table S1). Therefore, shared genotypes were included only once. In addition, one sample containing more than three null alleles was excluded from the dataset. Italian samples had 25% of successfully genotyped markers, probably due to low amount of DNA obtained from fecal samples and were thus excluded from the following analyses. Accordingly, a final data set consisted of 82 unique multilocus genotypes (Croatia n = 31, Czech Republic n = 4, Sierra Espuña n = 17, Almeria captivity n = 15, La Palma n = 15).

### Hardy‐Weinberg equilibrium and within-population genetic diversity

The fifteen microsatellite loci yielded a total of 76 alleles, ranging from 2 (BM302, MAF70 and TGLA073) to 9 (INRA040), with a mean number of alleles per locus of 5.07 (Supplementary Table S2). The PIC values ranged from 0.32 to 0.82, with an average of 0.57 (Supplementary Table S2). No evidence of scoring errors due to stuttering or large allele dropout was found in the whole data set. According to MICRO-CHECKER, the presence of null alleles was suggested in one locus/population combination (BM143 in Croatia) with the frequency of null alleles equal to 0.096 (Supplementary Table S3). However, Croatian population showed no departure from Hardy–Weinberg equilibrium (HWE) at this locus. The allelic richness varied from 1.93 (Almeria, Spain) to 3.67 (Sierra Espuña, Spain) (Table 1). Private alleles were observed in each population, with a total of 24 private alleles detected in four populations. The highest number of private alleles (9) was observed in captive population from Almeria. Observed heterozygosity values were between 0.358 in Almeria and 0.541 in Sierra Espuña, while unbiased expected heterozygosity ranged from 0.337 in Almeria to 0.564 in Sierra Espuña (Table 1).

**Table 1:** Genetic diversity assessed from fifteen microsatellite markers in four European aoudad populations.

Locality	Country	n	N_e_ (95% CI)	N_av_	N_ar_	N_pr_	N_par_	H_o_	H_e_	*f*
Mosor	Croatia	31	5.4 (3.1–10.0)	2.53 (1.13)	2.48 (1.07)	6	0.36 (0.47)	0.40 (0.26)	0.42 (0.27)	0.07
Sierra Espuña	Spain	17	36.0 (16.4–435.2)	3.67 (1.11)	3.61 (1.07)	6	0.39 (0.59)	0.54 (0.25)	0.58 (0.20)	0.07
La Palma	Spain	15	21.7 (9.3–178.8)	3.07 (1.03)	3.06 (1.02)	3	0.23 (0.43)	0.53 (0.20)	0.56 (0.18)	0.06
Almeria	Spain	15	22.8 (3.2–∞)	1.93 (0.59)	1.93 (0.59)	9	0.61 (0.83)	0.36 (0.23)	0.35 (0.21)	− 0.03

n = number of successfully genotyped individuals per sampling population, N_av_ = mean number of alleles per locus, N_ar_ and N_par_ = rarefied allelic and private allelic richness (smallest sample size = 28 alleles, as explained in materials and methods section), N_pr_ = total number of private alleles, H_o_ = observed and H_e_ = expected heterozygosity, *f* = estimator of the inbreeding coefficient (none of the values were statistically different from 0, with *P* < 0.05).

Linkage disequilibrium was observed for: 14 pairs of loci in Croatia, four pairs in Sierra Espuña, and for one pair of loci in La Palma and Almeria. After applying FDR corrections, linkage disequilibrium was significant only for MM12/SR-CSRP12 and TGLA073/SR-CSRP12 in Croatia. The multilocus value of the *f* estimator ranged between − 0.029 (Almeria) to 0.070 (Sierra Espuña) with a mean positive value of 0.302 ± 0.057.

Estimated effective population size was the smallest in Croatian population reflecting the small number of founding individuals, and the largest in Sierra Espuña.

Mean proportion of shared alleles between all individuals within each population was: 0.56 in Sierra Espuña, 0.57 in La Palma, 0.66 in Croatia, 0.75 in Almeria and 0.85 in the Czech population.

### Genetic differentiation and structure

Global F_ST_ value was 0.296 (95% CI = 0.249–0.347). The lowest pairwise F_ST_ value was observed between populations from Czech and Sierra Espuña (F_ST_ = 0.109), while the highest value was found between Croatian and Almeria populations (F_ST_ = 0.459) (Table 2). Global and all pairwise F_ST_ values were significantly different from zero (*P* < 0.01).

**Table 2:** Pairwise values of genetic differentiation (F_ST_) between European aoudad populations based on 15 microsatellite loci.

Population	Locality	Country	PO1	PO2	PO3	PO4
PO1	Mosor	Croatia				
PO2	Area near the city of Plzeň	Czech	0.143			
PO3	Sierra Espuña	Spain	0.157	0.109		
PO4	La Palma	Spain	0.282	0.215	0.142	
PO5	Almeria	Spain	0.459	0.444	0.385	0.393

All pairwise F_ST_ values between populations were significant at *P* < 0.01.

**Figure 3:**
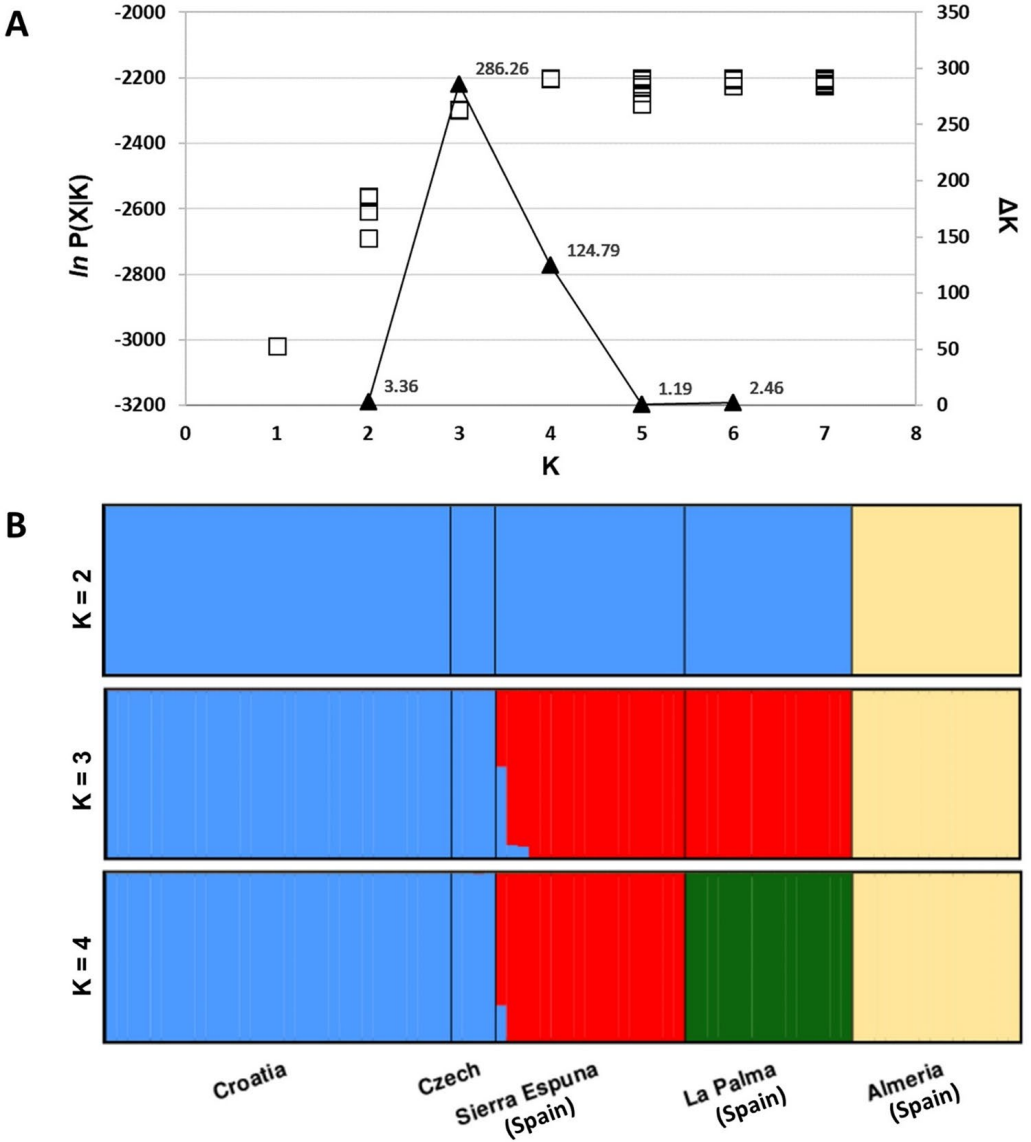
(**A**) The choice of the most likely number of clusters (K) inferred from STRUCTURE model based clustering: *ln* P(X|K) values (presented as the white squares) for each of the ten independent runs for each K and ΔK values (presented as the black triangles) for each K (shown on logarithmic scale) based on the second order rate of change of the likelihood function with respect to K. (**B**)Genetic structure of five European aoudad populations as estimated by the STRUCTURE for K = 2 to K = 4. Each individual is represented by a single vertical line partitioned in K‐colored segments, which correspond to the individual’s estimated proportion of membership in K clusters. Black lines delineate populations that are labeled below the figure. The figure was modified using Microsoft Excel 2016 and Microsoft PowerPoint 2016 (Microsoft Corporation, Redmond, WA, USA).

Among STRUCTURE runs, the highest ΔK value was observed for K = 3, followed by that for K = 4 (Fig. 3). Captive population from Almeria was unequivocally discriminated from the individuals belonging to free-ranging populations at each presented K value. After accounting for this major split, our results suggest the presence of two clusters in the four free-ranging populations surveyed. The first cluster is composed of Croatian and Czech populations, indicating that they belong to the same ancestral population. The second cluster includes Sierra Espuña and La Palma populations, while at K = 4, they appear as separate.

## Discussion

While this research aimed to present the genetic structure of all aoudad populations in Europe, we were not able to obtain any results for the Italian population due to the very low quality of DNA sampled from fecal samples.

Four distinct haplotypes found in European aoudad populations indicate that aoudads have been introduced in Europe from at least four maternal lineages. Sierra Espuña is the only population in which more than one haplotype was detected. The presence of multiple maternal lineages in this population, along with largest estimate of effective population size of all sampled populations, indicates that it was established by females with at least three different mtDNA haplotypes. Higher genetic diversity of Sierra Espuña population than of all other European populations is further supported by its lowest mean proportion of shared alleles (0.56) between individuals from this population and the largest estimated effective population size of 36.

Sierra Espuña population is known to have originated from individuals from the Frankfurt Zoo in Germany and the Ain Sebad Zoo in Casablanca, Morocco^10,25^. There is no available data on subspecies/lineages of aoudads used in introductions to Sierra Espuña (or in preceding introductions to Frankfurt and Ain Sebad Zoos), nor published mtDNA sequences from original populations that would allow for identification of subspecies/lineages present in this population, but variability of detected haplotypes might reflect introductions from multiple distinct lineages. This is especially valid for haplotype Amle04, which differs by at least 46 mutations from other haplotypes detected in this population, indicating its origin from evolutionary distinct units. Reported numbers of mutations between haplotypes from same subspecies of ungulates are usually lower. For example, in the study of chamois (*Rupicapra* sp.)^26^, native European mountain ungulate, D-loop haplotypes from the same subspecies were up to 15 mutations apart. In its native range in North Africa, six aoudad subspecies have been described, based on their distribution and morphological differences in coat color and horns^27^. Derouiche et al.^22^ aimed to further evaluate the validity of the subspecies and determine the geographical limits of the valid ones using mitochondrial cytochrome *b* gene. Based on the haplotypes obtained, their results indicated a deep Mediterranean-Saharan genetic break in the species, suggesting the presence of two highly distinct evolutionary lineages.

All analyzed aoudad populations in Europe were only recently introduced, with relatively small number of founding individuals, so it is expected that their genetic diversity is low in comparison to the viable free mating populations of native ungulates. This is confirmed by the number of detected alleles within all populations (N_ar_ was less than 3.61 in all analysed populations) and mean proportions of shared alleles within all populations (> 0.55) which confirm their poor genetic diversity. The theoretical expectations for the loss of allelic variation after bottlenecks are described by Nei et al.^28^ who showed that critical factors determining this loss are the size of founding population and the growth rate of a newly established population. They indicated that the average number of alleles per locus will be more sensitive to founding population size than heterozygosity. This is illustrated in most of sampled aoudad populations, in which allelic richness is substantially low (Table 1), while deficit of heterozygotes is not significant. Similarly low levels of genetic variability were previously reported for native European mountain ungulates (e.g. Šprem and Buzan^29^, for chamois), where it was attributed to isolation and poor management practices. Largest number of detected alleles and the highest observed heterozygosity in Sierra Espuña population is another indication of the highest genetic variability in this population, supporting the theory of multiple origins.

In all presented STRUCTURE results, Croatian and Czech populations cluster in the same genetic cluster, indicating joined origin. Among three populations from Spain, Almeria population shows as genetically distinct from others in results from K = 2 to K = 4, while Sierra Espuña and La Palma diverge only at K = 4. It is worth pointing out that the population from Almeria originated from a founding couple captured in the Atlantic Sahara (south of Morocco) back in 1975; so that it presumably belongs to the Saharan aoudad subspecies, while the other ones analyzed in our study might be of admixed origin, based on the fact that it is a population formed by individuals coming from the Casablanca Zoo in Morocco (probably belonging to the Atlas subspecies, the most abundant subspecies in that country), and from the Frankfurt Zoo, of unknown origin. In addition, Almeria population had the second highest mean proportion of shared alleles (0.75), lower only than the small Czech population.

Management of European aoudad populations varies between countries and the views change with new insights of their coexistence with native species and ecosystems^30^. Implications of detected low genetic diversity on management of these populations can be discussed only after management goals are clearly defined. Commonly accepted practice is to increase genetic variability of introduced species by introducing more individuals with different genetic background. The effects of such practices were reviewed by Dlugosch and Parker^31^ who quantitatively summarized the genetic diversity data available at the time for 80 introduced or invasive species of animals, plants and fungi. Their review shows that multiple introductions had only small positive effects on diversity on average, disapproving the argument that multiple introductions have been critical in providing genetic rescue from severe and deleterious founder effects in most cases. According to this review, increases in genetic diversity caused by multiple introductions and/or gene flow occur after many decades, during which time the range expansion of the founder population is successful. Later introductions of genetically distinct individuals can increase genetic diversity through admixture, and the strength of this effect will depend on the reproductive rate of the individuals from different “waves” of introductions.

## Materials and methods

### Sampling and study area

From 2016 to 2018, we collected 92 samples of *A. lervia* individuals from Croatia (Mosor Mountain; 43°31′23″N 16°38′9″E), Italy (Beigua Regional Park in the province of Savona; 44°25′58″N 8°32′55″E) and Spain (Sierra Espuña, Murcia province; 37°51′10″N 1°32′34″W, and La Palma island; 8°44′41″N 17°53′08″W). These populations represent the overall distribution range of free-ranging aoudad populations in Europe. Also, we obtained samples from a captive population from the Czech Republic (near the city of Plzeň; 49°53′17″N 13°19′12″E) and those of the Saharan aoudad, *A. l. sahariensis*, in Almeria, Spain; 36°49′48″N 2°24′29″W.

In Croatia, 34 tissue samples were collected during regular hunting. In the Czech Republic, four blood samples were collected from live aoudad because they are protected and not hunted^17^. Since the aoudad population in Italy is small and we were not able to collect any tissue samples, we collected seven fresh faeces samples. In Spain 47 tissue samples were collected from three locations: Sierra Espuña n = 17, La Palma n = 15, during regular hunting, and from captivity in Almeria n = 15. Geographic locations of the sampled populations are presented in Fig. 1.

Sampling was done according to the Ethical and Welfare Standards presented in the (Official Gazette of the Republic of Croatia 102/2017), Regulation on the Protection of Animals Used for Scientific Purposes (Official Gazette of the Republic of Croatia 55/13), with the approval of the Bioethical Committee for the Protection and Welfare of Animals of the University of Zagreb Faculty of Agriculture (UR.BR. 251-71-29-02/19-21-1). All the research was done in accordance with arrive guidelines.

### Laboratory procedures

Total genomic DNA from tissue samples was extracted using the Promega Wizard Genomic Purification Kit following the manufacturer’s instructions. From faeces samples, DNA extraction was done using a liquid handling robot (Hamilton Starlet) and optimization by Applied Biosystems MagMAX DNA extraction Kit. All individuals were genotyped using fifteen polymorphic microsatellite loci: BM143, BM302, BM415, BM1443, BM1818, ETH225, ILSTS030Q, INRA005, INRA040, MAF70, MB25, MM12, SR-CSRP12, SR-CSRP24, TGLA073^32^. For the fluorescent labeling of PCR fragments, we used the M13-tailed primer method^33^. Singleplex PCRs were performed in a final 8 µL reaction volumes consisting of 1 × GoTaq G2 Hot Start Colorless Master Mix (Promega), 0.2 µM of each reverse and M13 primer, 0.05 µM of forward primer and 1 µL of template DNA. Information about PCR hybridization temperatures of all loci and thermocycler programme is given in Beja-Pereira et al.^32^. PCR products were analyzed on an ABI 3730 Genetic Analyser with GeneScan 350 ROX internal size standard (Applied Biosystems) by Macrogen (South Korea). GeneMapper v3.2. (Applied Biosystems) was used for scoring alleles.

Mitochondrial control region was amplified using tRNAPro and tRNAPhe primers^34^, following the PCR protocol given in Mereu et al.^34^. The PCR products were sequenced in forward direction using an ABI 3730 automated DNA sequencer (Applied Biosystems). Electropherograms were visually inspected using Applied Biosystems SEQSCAPE software and the sequences were trimmed to 767 bp which could be unequivocally called.

### Mitochondrial sequence analysis

The resulting sequences were aligned with two previously published sequences of the aoudad complete mitogenome (GenBank accession numbers FJ207522 and EF466060) using the ClustalW algorithm implemented in MEGA X^35^. The presence of tandem repeats in the 5′ part of the control region led to ambiguity in the position of the gaps and for this reason we excluded the ambiguous region from final sequences used in further analyses. A final alignment was obtained by merging the two trimmed fragments at positions 15,448–15,568 and 15,730–16,214 with respect to the GenBank sequence (accession number FJ207522) and consisted of 606 nucleotides. DnaSP v.6 was used to estimate number of haplotypes, haplotype (*Hd*) and nucleotide (*π*) diversity^36^. The evolutionary relationships between the haplotypes were analyzed by a median-joining (MJ) network^37^ which was constructed using PopART^38^. The parameter ε was set to zero (default) to obtain a sparse spanning network.

### Genetic diversity

For all population based analyses we used only genotypes from populations with more than 10 samples (Croatian and all four Spanish populations). MICRO-CHECKER 2.2.3^39^ was used to check amplified microsatellite genotypes for large allele dropout, scoring errors due to stuttering and the presence of null alleles. Null allele frequencies were then estimated for each locus and population using the expectation maximization algorithm^40^ implemented in FreeNA^41^. Polymorphism Information Content (PIC) was calculated for each microsatellite marker per population using CERVUS v.3.0.7^42^. GENETIX v.4.05^43^ was used to estimate the mean number of alleles, the observed heterozygosity (H_o_), expected hererozygosity (H_e_) and *f* estimator of F_IS_^44^ per locus and population. Linkage disequilibrium was tested among all pairs of loci in each population using a permutation procedure (n = 1000) using GENETIX v.4.05. Departures from Hardy–Weinberg equilibrium (HWE) were tested for each population using the score tests for heterozygote deficiency implemented in GENEPOP v.4.7.2^45^. Allelic richness [N_ar_(g)] and private allelic richness [N_par_(g)] were estimated using a rarefaction procedure implemented in HP-RARE^46^, where g represents the minimum number of alleles observed at a locus in one of the populations (i.e., twice the number of genotypes). The minimum number of genes in analysed populations was 28 (since locus locus INRA005 failed to aplify for one individual in the Almeria population), so this was used as a basis for rarefraction.

We estimated effective population size (Ne) for each population using the linkage disequilibrium method^47^, as implemented in NEESTIMATOR 2.01^48^. As suggested by Waples and Do^49^, we excluded alleles with frequencies below 0.02 in order to avoid bias caused by rare alleles.

Finally, to estimate the genetic similarity of individuals within populations, we estimated the mean proportion of shared alleles between all individuals within each population using custom script in Microsoft Excel 2016 (Microsoft Corporation, Redmond, WA, USA, https://www.microsoft.com/en-ca/microsoft-365/excel).

### Genetic differentiation and structure

We estimated overall and pairwise genetic differentiation with the *θ* estimator of F_ST_^44^ and determined their statistical significance by 1000 permutations in FSTAT. Finally, to infer the number of ancestral populations among non-native aoudad populations, we identified genetic structure using a model-based clustering method implemented in STRUCTURE v. 2.3.4^50^. Ten runs per cluster (K), with K ranging from 1 to 7, were carried out with 10^6^ iterations after a burn-in period of 10^5^ iterations. We considered no admixture model and uncorrelated allele frequencies. The most likely number of clusters (K) was estimated by estimation of the ΔK statistic that reflects the rate of change of log-likelihood values between sets of runs with successive K values, using STRUCTURE HARVESTER^51^. To account for label switching between results of different runs with same K, results from 10 runs with selected K were combined using CLUMPP v.1.1.2^52^. Results were displayed graphically using DISTRUCT v.1.1^53^ and modified with Microsoft PowerPoint 2016 (Microsoft Corporation, Redmond, WA, USA, https://www.microsoft.com/en-ca/microsoft-365/powerpoint).

## Supplementary Information

Supplementary Information 1.

Supplementary Information 2.

Supplementary Information 3.

## Data Availability

Data generated and analysed during this study are included in the Supplementary Information file and are available in the GenBank repository (Accession Nos. MW349820–MW349823).
