# Supplementary material for: Population structure and genetic diversity of non-native aoudad populations
Source: Sci Rep. 2021 Jun 10;11:12300. doi: 10.1038/s41598-021-91678-2 (PMC8192935; doi:10.1038/s41598-021-91678-2)
Supplement: Supplementary file 1 — Supplementary Information 1. [file 41598_2021_91678_MOESM1_ESM.docx]

| Locus | Single-locus PI(unbiased) | Multi-loci PI(unbiased) |
| --- | --- | --- |
| BM1818 | 1.43E-01 | 1.43E-01 |
| ILSTS030Q | 1.43E-01 | 2.05E-02 |
| MM12 | 1.49E-01 | 3.06E-03 |
| ETH225 | 1.59E-01 | 4.86E-04 |
| SR-CSRP24G | 2.26E-01 | 1.10E-04 |
| INRA040 | 2.61E-01 | 2.87E-05 |
| BM143 | 2.74E-01 | 7.85E-06 |
| TGLA073S | 3.73E-01 | 2.92E-06 |
| SR-CSRP12G | 3.73E-01 | 1.09E-06 |
| MAF70S | 3.73E-01 | 4.06E-07 |
| MB25 | 4.95E-01 | 2.01E-07 |
| BM1443 | 7.32E-01 | 1.47E-07 |
| BM415 | 1.00E+00 | 1.47E-07 |
| INRA005 | 1.00E+00 | 1.47E-07 |
| BM302 | 1.00E+00 | 1.47E-07 |
| Mean | 4.47E-01 | 1.12E-02 |
| SD | 0.33 | 0.04 |

**Supplementary Table S1.** Unbiased probability of identity (PI) computed using Gimlet v.1.3.3 for 33 aoudads (*Ammotragus lervia*) from Croatian population based on 15 microsatellite loci.

The PI is calculated for each locus [single-locus PI(unbiased)] and overall by multiplying sequentially the PI value over loci [multi-loci PI(unbiased)]. PI values are ranked in increasing order of single-locus PI(unbiased) values.
