# Supplementary material for: Population structure and genetic diversity of non-native aoudad populations
Source: Sci Rep. 2021 Jun 10;11:12300. doi: 10.1038/s41598-021-91678-2 (PMC8192935; doi:10.1038/s41598-021-91678-2)
Supplement: Supplementary file 2 — Supplementary Information 2. [file 41598_2021_91678_MOESM2_ESM.docx]

| No. | Locus | n | N_a_ | Size Range | PIC |
| --- | --- | --- | --- | --- | --- |
| 1 | BM143 | 82 | 7 | 115 - 135 | 0.704 |
| 2 | BM302 | 82 | 2 | 173 - 177 | 0.319 |
| 3 | BM415 | 82 | 5 | 128 - 163 | 0.477 |
| 4 | BM1443 | 82 | 6 | 136 - 167 | 0.490 |
| 5 | BM1818 | 82 | 8 | 268 - 290 | 0.822 |
| 6 | ETH225 | 82 | 5 | 171 - 194 | 0.612 |
| 7 | ILSTS030Q | 82 | 5 | 183 - 195 | 0.728 |
| 8 | INRA005 | 80 | 3 | 178 - 197 | 0.248 |
| 9 | INRA040 | 82 | 9 | 191 - 225 | 0.780 |
| 10 | MAF70 | 82 | 2 | 153 - 155 | 0.344 |
| 11 | MB25 | 82 | 6 | 209 - 255 | 0.596 |
| 12 | MM12 | 82 | 5 | 107 - 127 | 0.572 |
| 13 | SR-CSRP12 | 82 | 5 | 109 - 123 | 0.650 |
| 14 | SR-CSRP24 | 81 | 6 | 166 - 186 | 0.768 |
| 15 | TGLA073 | 82 | 2 | 102 - 104 | 0.374 |
|  | Mean |  | 5.067 |  | 0.566 |

**Supplementary Table S2.** Allelic diversity of 15 microsatellite loci scored in five European aoudad populations. n - number of successfully genotyped individuals; N_a_ - total number of alleles per locus; PIC - polymorphism information content.
