# Supplementary material for: Population structure and genetic diversity of non-native aoudad populations
Source: Sci Rep. 2021 Jun 10;11:12300. doi: 10.1038/s41598-021-91678-2 (PMC8192935; doi:10.1038/s41598-021-91678-2)
Supplement: Supplementary file 3 — Supplementary Information 3. [file 41598_2021_91678_MOESM3_ESM.docx]

| **Population** | **BM143** | | **BM302** | | **BM415** | | **BM1443** | | **BM1818** | | **ETH225** | | **ILSTS030QQ** | | **INRA005** | |
| --- | --- | --- | --- | --- | --- | --- | --- | --- | --- | --- | --- | --- | --- | --- | --- | --- |
|  | *f* | Null | *f* | Null | *f* | Null | *f* | Null | *f* | Null | *f* | Null | *f* | Null | *f* | Null |
| Mosor, Croatia | 0.329 | 0.096 | / | 0.001 | / | 0.001 | 0.478 | 0.095 | **0.209** | 0.091 | 0.038 | 0.006 | 0.162 | 0.050 | / | 0.001 |
| Sierra Espuña, Spain | 0.017 | 0.000 | 0.458 | 0.109 | 0.375 | 0.107 | 0.079 | 0.021 | 0.177 | 0.038 | -0.195 | 0.000 | 0.028 | 0.020 | 0.129 | 0.000 |
| La Palma, Spain | 0.200 | 0.055 | -0.308 | 0.000 | -0.167 | 0.000 | 0.311 | 0.120 | 0.139 | 0.045 | 0.139 | 0.000 | 0.100 | 0.013 | 0.650 | 0.141 |
| Almeria, Spain | 0.152 | 0.015 | -0.167 | 0.000 | -0.037 | 0.000 | / | 0.001 | -0.120 | 0.000 | -0.217 | 0.000 | / | 0.001 | 0.032 | 0.000 |
|  |  |  |  |  |  |  |  |  |  |  |  |  |  |  |  |  |
|  | **INRA040** | | **MAF70S** | | **MB25** | | **MM12** | | **SR-CSRP12G** | | **SR-CSRP24G** | | **TGLA073S** | | **All loci** | |
|  | *f* | Null | *f* | Null | *f* | Null | *f* | Null | *f* | Null | *f* | Null | *f* | Null | *f* | Null |
| Mosor, Croatia | 0.095 | 0.036 | 0.163 | 0.049 | -0.017 | 0.000 | 0.012 | 0.000 | -0.177 | 0.000 | -0.213 | 0.000 | -0.044 | 0.000 | 0.069 | 0.028 |
| Sierra Espuña, Spain | -0.007 | 0.000 | 0.484 | 0.134 | -0.167 | 0.000 | 0.074 | 0.025 | 0.232 | 0.073 | -0.114 | 0.000 | 0.130 | 0.032 | 0.070 | 0.037 |
| La Palma, Spain | 0.004 | 0.000 | -0.077 | 0.000 | -0.235 | 0.000 | -0.112 | 0.000 | -0.112 | 0.000 | 0.203 | 0.064 | 0.364 | 0.111 | 0.060 | 0.037 |
| Almeria, Spain | -0.167 | 0.000 | 0.548 | 0.165 | -0.167 | 0.000 | / | 0.001 | -0.308 | 0.000 | 0.030 | 0.000 | -0.120 | 0.000 | -0.029 | 0.012 |

**Supplementary Table S3.** Null allele frequencies (Null) and *f* estimator of F_IS_ per sample and locus. Significant deviations from Hardy-Weinberg equilibrium, at the 0.05 level after FDR correction, are in bold.
